# Diseases of the Stomach and Intestines

**Published:** 1907-03

**Authors:** 


**Diseases of the Stomach and Intestines.** By **Boardman Reed,** M.D., Late Physician in Chief to the Samaritan Hospital, Philadelphia. Second edition. Illustrated. Octavo, pp. 1021. New York: E. B. Treat & Co. 1907. (Price, $5.00).

It is less than two years since the first edition of this book was published and in that time its popularity has been demonstrated; hence it is not surprising a new edition has been found necessary. Special attention has been given in the newer work to the more important advances and therapeutic improvements in the field covered, and much new material has been added. A fact which must be pleasing to the author is that several matters which, in the first edition. were adversely criticised, have since that time become generally recognised and accepted. Particularly is this demonstrated in the recognition of eye strain as a cause of gastrointestinal symptoms; dependence on hygiene and diet as well as electric and other mechanical measures in preference to routine drug giving, and the recommendation of extreme caution in the use of tuberculin. The fact that his note of warning regarding the latter has been generally accepted and followed must be particularly gratifying to Dr. Reed. The book is an excellent one for the general practitioner and it may be followed with much benefit in daily work. It will prove of great value to the younger men of the profession.

